# Immunological phenotype in asthma and its impact on long-term renal outcomes

**DOI:** 10.1038/s41598-025-18035-5

**Published:** 2025-10-21

**Authors:** Wang Chung Kwok, Terence Chi Chun Tam, James Chung Man Ho, David Chi Leung Lam, Isaac Sze Him Leung, Mary Sau Man Ip, Desmond Yat Hin Yap

**Affiliations:** 1 Division of Respiratory Medicine, Department of Medicine, Queen Mary Hospital, The University of Hong Kong, Hong Kong, China; 2 Department of Statistics, The Chinese University of Hong Kong, Hong Kong, China; 3 Division of Nephrology, Department of Medicine, Queen Mary Hospital, The University of Hong Kong, Room 301, New Clinical Building, 102 Pokfulam Road, Hong Kong, China

**Keywords:** Asthma, Phenotype, Renal impairment, Chronic kidney disease, Asthma, Chronic kidney disease

## Abstract

Asthma is associated with both airway and systemic inflammation as well as non-respiratory adverse outcomes. However, data regarding its impact on long-term renal outcomes is lacking. We classified all asthma patients who were followed at Queen Mary Hospital in 2017 into eosinophilic or non-eosinophilic phenotypes based on their highest blood eosinophil counts (BEC) during stable state in the year (≥ 300 or < 300 cells/mm^3^ respectively) and prospectively evaluated their clinical outcomes in the subsequent 5 years. The relationship between patient phenotypes and the long-term renal outcomes were assessed. Five hundred and four asthma patients with baseline Stage 1 to 3 chronic kidney disease were included [296 (58.7%) and 208 (41.3%) in eosinophilic and non-eosinophilic groups respectively]. Among patients with baseline renal function at CKD stage 1 to 3, one hundred and four patients (20.6%) had renal progression in this cohort (56 patients (26.9%) vs. 48 patients (16.2%) in the non-eosinophilic and eosinophilic groups respectively). Patients with non-eosinophilic asthma showed increased risks of renal progression over 5 years of follow-up [adjusted odds ratio (aOR) 2.615, 95% CI 1.151–5.942 p = 0.022] and more rapid eGFR decline (−4.29 ± 3.48 mL/min/1.73m^2^/year vs. −3.48 ± 3.07 mL/min/1.73m^2^/year, p = 0.007) than those with eosinophilic phenotype. Patients who developed renal progression had higher risk of death [adjusted hazard ratio (aHR) 1.614 (95% CI 1.041–2.502); p = 0.032]. Progressive renal function deterioration is prevalent amongst asthma patients, and those with non-eosinophilic phenotype are at risk of renal progression.

**Supplementary Information:**

The online version contains supplementary material available at 10.1038/s41598-025-18035-5.

## Introduction

In order to provide personalized treatment, phenotyping in respiratory diseases is crucial. In asthma, patients with different phenotypes have distinct clinical features, as well as different risks of deterioration^1–3^. The phenotypes also affect the treatment of choice, including sensitivity to glucocorticoids and biologics that target at different inflammatory mediators^4–6^. Blood eosinophil count (BEC) is one of the simple and readily available tools to categorize patients into different phenotypes, based on the inflammatory pathways. Patients with baseline BEC ≥ 300 cells/mm^3^ is classified to have eosinophilic phenotype^7–10^, which is hallmarked by Th2 inflammation mediated by T helper 2 cells and related cytokines including interleukin (IL)-4, IL-5 and IL-13^11,12^. Patients with non-eosinophilic asthma, also known as type-2 low or non-type 2 asthma, is characterized by normal sputum and peripheral blood eosinophil counts, low fractional exhaled nitric oxide (FeNO), yet with persistent symptoms and airflow limitation and poor response to corticosteroids^13^.

Apart from respiratory tract involvement, asthma is also reported to have systemic inflammation and associations with non-respiratory adverse outcomes. Studies found that systemic inflammatory markers were elevated in patients with non-allergic asthma and obese patients with asthma^14^. Epidemiological studies demonstrated deleterious systemic cardiovascular effects associated with the asthma, including atherosclerotic cardiovascular events, atrial fibrillation and hypertension^15^. In retrospective studies, persistent asthma was found to be associated with an increased risk of chronic kidney disease (CKD)^16,17^. A bidirectional Mendelian randomization study also suggested increased estimated glomerular filtration rate (eGFR) to be associated with reduced risk of late-onset asthma^18^.

Recognizing the link between asthma and CKD, however, is not adequate, as asthma is a persistent disease with substantial patient heterogeneity. Owing to the difference in pathophysiological mechanisms, the phenotype of asthma will not only influence the pharmacotherapy of choice and its treatment outcomes, but disease prognosis and spectrum of non-respiratory adverse events. Based on these backgrounds, this study was set forth to investigate the relationship between asthma phenotypes and adverse renal outcomes. The results will help identify asthma patients at risk of renal progression, and hence institute regular kidney function monitoring and treatment for these individuals.

## Methods

We classified all asthma patients with underlying renal function at CKD stage 1 to 3 who were followed at Queen Mary Hospital (QMH) in 2017 into eosinophilic or non-eosinophilic phenotypes based on their highest blood eosinophil counts (BEC) during stable state in the year (BEC ≥ 300 or < 300 cells/mm^3^ respectively)^7–10^, and prospectively evaluated their clinical outcomes in the subsequent 5 years. QMH is a tertiary referral centre and a major teaching hospital in Hong Kong, with a catchment population of approximately 500,000. Patients who were lost to follow-up or without baseline renal function tests; as well as those with underling CKD stage 4 to 5 were excluded from analysis. Clinical data and relevant laboratory results were systematically entered into the electronic patient records (ePR) of the Hospital Authority of Hong Kong and later retrieved for analysis. The study was approved by the Institutional Review Board of the University of Hong Kong and Hospital Authority Hong Kong West Cluster (IRB number: UW 23-356).

Regular use of long-acting beta-agonists (LABA), long-acting muscarinic antagonists (LAMA), leukotriene antagonists (LTRA), inhaled corticosteroids (ICS) and theophylline were defined as continuous use of the medication for at least 6 months before recruitment into this study. The dose of ICS was defined using the global initiative for asthma (GINA) recommendation^19^. Baseline lung function test was performed at clinical stable state that was at least 3 months apart from the last exacerbation, and within 12 months from the case recruitment.

According to the medical record from ePR, the renal function was measured every 8 to 32 weeks in this study, with at least 3 measurements available for each patient.

The primary outcome was the development of renal progression upon follow-up and was compared between patients with eosinophilic and non-eosinophilic asthma, among patients with underling CKD stage 1 to 3. Renal progression was defined as persistent drop in eGFR from baseline of more than 30 mL/min/1.73 m^2^ that persisted more than 12 months^20^. The stage of CKD was defined according to K/DOQI clinical practice guidelines for chronic kidney disease: evaluation, classification, and stratification definition^20^. The secondary outcomes included the predictors of development of renal progression and the survival of patients with or without renal progression.

### Statistical analysis

Categorical variables were expressed as frequency and proportion (percentage) and compared with Chi-square tests or Fisher’s Exact tests where appropriate. Continuous variables were expressed as mean (± standard deviation [S.D.]) and compared with Student’s t-tests or Mann Whitney U tests where appropriate. The relationships between patients with different asthma phenotypes and adverse renal outcomes were first assessed by univariate analysis, then followed by multi-variable analysis adjusted for covariates including age, sex, body mass index (BMI), smoking history, presence of cardiovascular comorbidities (hypertension, diabetes mellitus, hyperlipidemia, ischaemic heart disease, stroke, transient ischaemic attack and atrial fibrillation), baseline eGFR, ACE/ARB use, baseline FEV_1_, use of LABA/LAMA/ICS and their doses and other factors that were significantly different at baseline if appropriate. The relationships between different clinical parameters, as well as mortality among patients with or without renal progression, were compared by logistic regression. The rate of eGFR decline (as measured by the change in absolute eGFR from baseline to end of follow-up divided by follow-up in years) was compared by linear regression. Cox regression analysis was used to assess overall survival. Kaplan–Meier analysis was used to estimate the cumulative death rates and the stratified log-rank statistics to assess the effects of renal progression in the follow-up period with respect to the composite endpoint of death. Multicollinearity between covariates were assessed using the Variance Inflation Factor (VIF). The statistical significance was determined at the level of p < 0.05 at two-sided test. All the statistical analyses were done using the 28^th^ version of SPSS statistical package.

## Results

### Patient characteristics

A total of 533 patients with asthma were identified (Table 1). Among these patients, 504 of them had baseline renal function at CKD stage 1 to 3. Among the 504 patients with stage 1–3 CKD, 191 (37.9%) were males, with a mean age of 60.6 ± 19.8 years. 400 (79.4%) were never smokers. The mean FEV_1_ was 1.76 ± 0.77 L (66.3 ± 13.8% predicted). The mean baseline eGFR were 82.6 ± 22.7 mL/min/1.73m^2^ and 83.0 ± 21.5 mL/min/1.73m^2^ in patients with eosinophilic and non-eosinophilic asthma subgroups respectively. The median annual number of hospitalized asthma exacerbation in the follow up period were 0 [0–0.18] in the eosinophilic group and 0 [0–0.35] in the non-eosinophilic group respectively, p = 0.22. There were 63 (21.3%) and 46 (22.1%) patients who had bacterial pneumonia in the eosinophilic and non-eosinophilic groups respectively, p = 0.83. There were 24 (8.1%) and 18 (8.7%) patients who had viral lower respiratory tract infections with hospitalization in the eosinophilic and non-eosinophilic groups respectively, p = 0.83.

**Table 1:** Baseline demographic and clinical characteristics of patients with eosinophilic and non-eosinophilic asthma with baseline CKD stage 1 -3.

	Non-eosinophilic asthma (n = 208)	Eosinophilic asthma (n = 296)	Whole cohort (n = 504)	p-values^
Age (years)	62.4 ± 21.1	59.4 ± 18.8	60.6 ± 19.8	0.100
Sex				< 0.001*
Male	57 (27.4%)	134 (45.3%)	191 (37.9%)	
Female	151 (72.6%)	162 (54.7%)	313 (62.1%)	
Smoking status				0.230
Non-smoker	171 (82.2%)	229 (77.4%)	400 (79.4%)	
Active smoker	12 (5.8%)	15 (5.1%)	27 (5.4%)	
Ex-smoker	25 (12.0%)	52 (17.6%)	77 (15.3%)	
BMI (kg/m^2^)	24.9 ± 5.6	23.9 ± 6.9	24.4 ± 6.3	0.155
Co-morbidities				
Hypertension	109 (52.4%)	119 (40.2%)	228(45.2%)	0.007*
Diabetes mellitus	45 (21.6%)	50 (16.9%)	95 (18.8%)	0.180
Hyperlipidemia	57 (27.4%)	63 (21.3%)	120 (23.8%)	0.112
Atrial fibrillation	12 (10.1%)	23 (7.8%)	44 (8.7%)	0.362
Stroke/TIA	24 (11.5%)	24 (8.1%)	48 (9.5%)	0.196
Ischemic heart disease	35 (16.8%)	50 (16.9%)	85 (16.9%)	0.985
Bronchiectasis	7 (3.4%)	20 (6.8%)	27 (5.4%)	0.096
Atopic dermatitis	70 (33.7%)	113 (38.2%)	183 (36.3%)	0.299
Allergic rhinitis	167 (80.3%)	249 (84.1%)	416 (82.5%)	0.264
Medication				
ACEI/ARB	93 (44.7%)	115 (38.9%)	208 (41.3%)	0.188
Inhaled corticosteroid				0.257
Low dose	29 (13.9%)	50 (16.9%)	79 (15.7%)	
Medium dose	59 (28.4%)	89 (30.1%)	148 (29.4%)	
High dose	81 (38.9%)	120 (40.5%)	201 (39.9%)	
Long acting beta-agonists	84 (40.4%)	147 (49.7%)	231 (45.8%)	0.040*
Long acting anti-muscarinic	39 (18.8%)	78 (26.4%)	117 (23.2%)	0.047*
Leukotriene antagonists	60 (28.8%)	101 (34.4%)	161 (31.9%)	0.211
Theophylline	63 (30.3%)	99 (33.4%)	162 (32.1%)	0.455
Baseline FEV_1_ (L)	1.68 ± 0.83	1.83 ± 0.73	1.76 ± 0.77	0.108
Baseline FEV_1_ (% predicted)	78.4 ± 23.3	78.5 ± 22.8	78.4 ± 22.9	0.988
Baseline FEV_1_ to FVC (%)	68.8 ± 13.5	64.5 ± 13.9	66.3 ± 13.8	< 0.010*
Bronchodilator reversibility (%)	8.5 ± 10.6	11.0 ± 25.6	10.1 ± 21.6	0.278
Bronchodilator reversibility (mL)	108 ± 138	139 ± 169	126 ± 159	0.172
Baseline neutrophil count(× 10^9^/L)	5.10 ± 2.59	4.78 ± 2.32	4.91 ± 2.44	0.160
Baseline lymphocyte count (× 10^9^/L)	1.69 ± 0.59	1.82 ± 0.75	1.78 ± 0.70	0.068
Baseline eosinophil count (× 10^12^/L)	27 ± 48	780 ± 709	490 ± 457	< 0.001*
Baseline HbA1C (%)	4.99 ± 2.07	4.67 ± 2.20	4.81 ± 2.15	0.184
Serum low-density lipoprotein (mmol/L)	2.03 ± 0.79	2.04 ± 0.87	2.04 ± 0.84	0.848
SII	627 ± 529	596 ± 601	606 ± 577	0.605
NLR	3.11 ± 2.10	3.12 ± 3.99	3.12 ± 2.43	0.964
Serum albumin level (g/L)	41.46 ± 4.63	41.60 ± 4.14	41.55 ± 4.31	0.768
eGFR (mL/min/1.73m^2^), mean ± SD	83.0 ± 21.5	82.6 ± 22.7	82.7 ± 22.2	0.143
Stage of CKD				0.245
Stage 1	83 (39.9%)	121 (40.9%)	204 (40.5%)	
Stage 2	94 (45.2%)	116 (39.2%)	210 (41.7%)	
Stage 3	31 (14.9%)	59 (19.9%)	90 (17.9%)	
Number of asthma exacerbation in past 12 months, mean ± SD	0.47 ± 0.83	0.54 ± 1.13	0.52 ± 1.03	0.406

*CKD* chronic kidney disease, *BMI* body mass index, *ACEI* angiotensin converting enzyme inhibitor, *ARB* angiotensin receptor blockers, *FEV1* forced expiratory volume in 1 s, *FVC* forced vital capacity, *eGFR* estimated glomerular filtration rates, *TIA* transient ischaemic attack, *SII* systemic immune-inflammation index (measured by neutrophil counts x platelet counts/lymphocyte counts); NLR, Neutrophil-to-lymphocyte ratio.

Data expressed as mean ± S.D.CKD stage by eGFR (mL/min/1.73m^2^): Stage 1: ≥ 90, Stage 2: 60–89, Stage 3: 30–49; Stage 4: 15–29, Stage 5: < 15.

### Asthma phenotype and long-term renal outcomes

Among patients with baseline renal function at CKD stage 1 to 3, one hundred and four patients (20.6%) had renal progression in this cohort (56 patients (26.9%) vs. 48 patients (16.2%) in the non-eosinophilic and eosinophilic groups respectively) (Table 2). Among the patients who developed renal progression, 6 progressed to end-stage kidney disease and required renal replacement therapy, 1 in the non-eosinophilic and 5 in the eosinophilic group (p = 0.238). At the last follow-up, 51 (10.1%) and 61 (12.1%) patients reached stage 4 and 5 CKD. Patients who had developed renal progression were older (78.9 years vs. 60.3 years, p < 0.001), with higher prevalence of hypertension (61.5 vs. 44.1%, p = 0.001), diabetes mellitus (28.8 vs. 19.1%, p = 0.029) and atopic dermatitis (46.2 vs 34.3%, p = 0.024), lower baseline FEV_1_ (1.51 ± 0.69 L vs 1.82 ± 0.78 L, p 0.003; 73.2 ± 21.2% vs 79.6 ± 23.1, p = 0.051), higher rates of ACEI/ARB use (65.4 vs. 37.1%, p < 0.001) and theophylline use (34.2 vs. 29.4%, p = 0.033) (Supplementary Table ST1 and ST2).

**Table 2:** Renal and Mortality Outcomes in patients with eosinophilic and non-eosinophilic asthma.

	Non-eosinophilic asthma (n = 208)	Eosinophilic asthma (n = 296)	Whole cohort (n = 504)	p-values^
Renal progression^+^	56 (26.9%)	48 (16.2%)	104 (20.6%)	0.022*
eGFR at 5 years^++^	61.5 ± 30.6	65.2 ± 29.2	63.7 ± 29.8	0.183
Rate of eGFR decline^++^	−4.29 ± 3.48	−3.48 ± 3.07	−3.82 ± 3.27	0.007*
Death^+^	45 (21.6%)	49 (16.6%)	94 (18.7%)	0.149

*eGFR* estimated glomerular filtration rates. *CKD* chronic kidney disease.

^+^Compared by logistic regression.

^++^Compared by linear regression.

Factors adjusted for age, sex, BMI, smoking history, presence of cardiovascular comorbidities, baseline eGFR, ACE/ARB use, baseline FEV_1_, use of LABA/LAMA/ICS and their doses.

*P < 0.05.

Data expressed as mean ± S.D

**Fig. 1:**
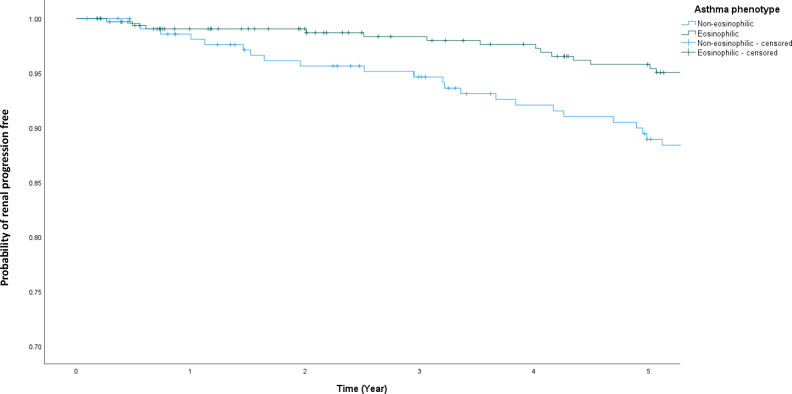
Renal Progression in patients with eosinophilic and non-eosinophilic asthma.

Patients with non-eosinophilic asthma showed worse renal progression-free survival than those with eosinophilic asthma (26.9 vs. 16.2% at 5 years, p = 0.003) (Fig. 1). Univariate and multivariate analysis showed that patients with non-eosinophilic asthma had increased risks of developing renal progression over 5 years of follow-up [odds ratio (OR) of 1.904 (95% confidence interval [CI] 1.232–2.941, p = 0.004); adjusted odds ratio (aOR) of 2.615 (95% CI 1.151–5.942, p = 0.022) after adjusted for age, sex, BMI, smoking history, presence of cardiovascular comorbidities, baseline eGFR, ACE/ARB use, baseline FEV_1_, use of LABA/LAMA/ICS and their doses.

Patients with lower baseline FEV_1_ (Litre), but not FEV_1_ as percentage predicted, had an increased risk of developing renal progression [OR of 1.805 (95% CI 1.190–2.740, p = 0.005) at univariate analysis, but not on multivariate analysis.

Multivariable analysis also revealed that patients with non-eosinophilic asthma had more rapid eGFR decline than those with eosinophilic asthma (−4.29 ± 3.48 mL/min/1.73m^2^/year vs—3.48 ± 3.07 mL/min/1.73m^2^/year, p = 0.007) (Table 2).

### Impact of renal progression on mortality in asthma patients

**Fig. 2:**
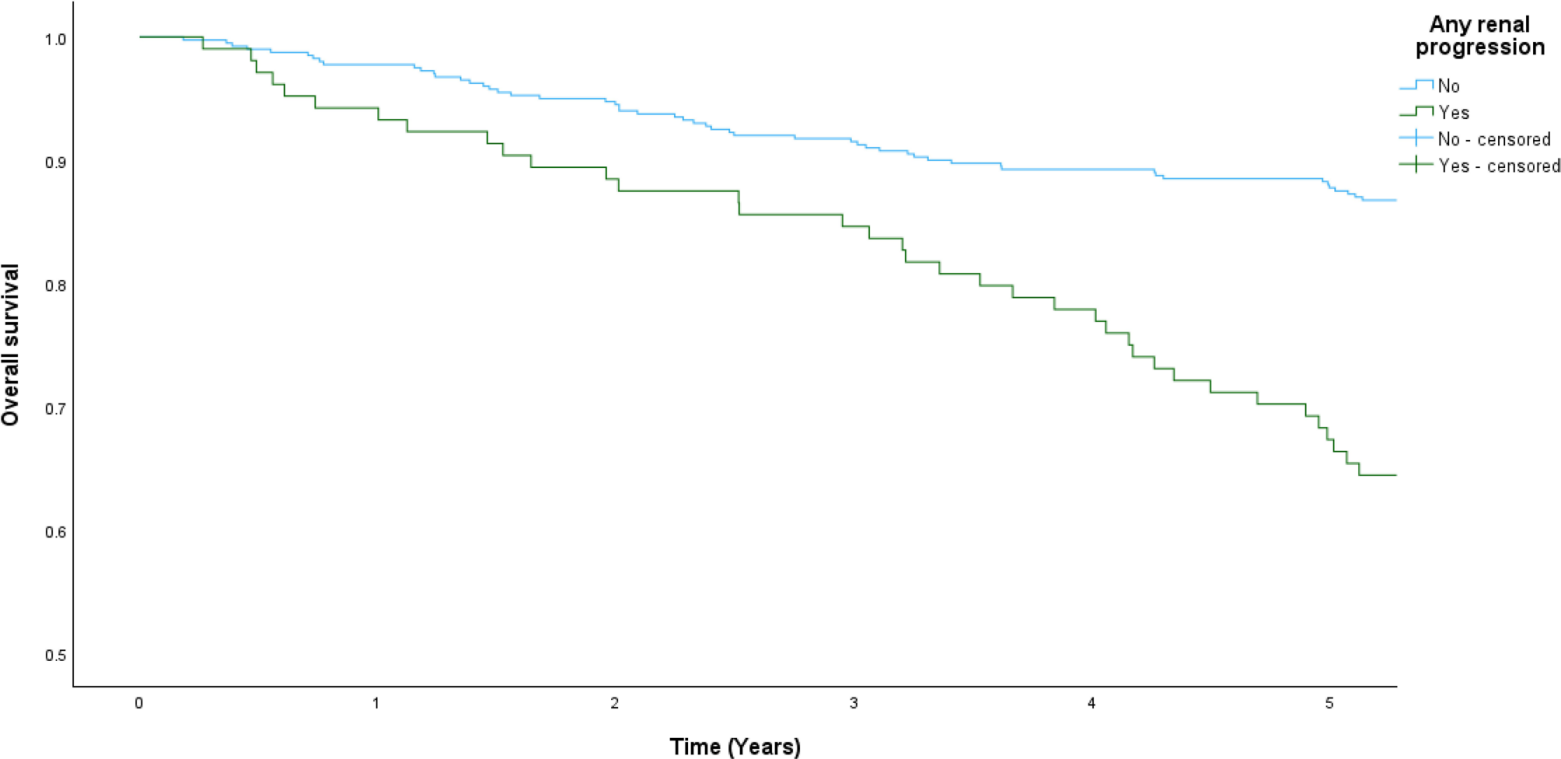
Survival of asthma patients with or without renal progression in the 5 years follow-up period.

Among patients with baseline renal function at CKD stage 1 to 3, 94(18.7%) patients died during a follow-up period of five years. 33 died of respiratory causes, 19 died of cardiovascular causes, 8 died of renal-related causes and 34 died of other causes. Patients who had renal progression showed worse overall survival than those without renal progression (41.5 vs. 15.9% at 5 years, p < 0.001) (Fig. 2). Univariate and multivariate analysis showed that patients who developed renal progression had increased risk of death [hazard ratio (HR) of 3.051 (95% CI 2.023–4.600; p < 0.001); adjusted hazard ratio (aHR) 3.381 (95% CI 1.093–10.461; p = 0.035) after adjusting for age, sex, BMI, smoking history, presence of cardiovascular comorbidities, baseline eGFR, ACE/ARB use, asthma phenotype, baseline FEV_1_, use of LABA/LAMA/ICS and their doses].The results were also significant in sub-group analysis that excluded patients who had RRT after renal progression, with aHR of 3.365 (95% CI 1.089–10.402; p = 0.035).

Rate of eGFR decline was also significantly associate with mortality, with OR of 1.169 (95% CI = 1.115–1.226, p < 0.001) and aOR of 1.207 (95% CI = 1.026–1.420, p = 0.023). This suggested that faster the eGFR decline, the higher the mortality.

### Subgroup analysis

There were 414 patients with baseline renal function of CKD stage 1 to 2. Non-eosinophilic phenotype had significantly increased risks of renal progression (aOR = 2.711, 95% CI = 1.109–6.629, p = 0.029). In the CKD stage 3 to 5 group, the same phenomenon could not be observed. The OR was 1.609 (95% CI = 0.596–4.347, p = 0.348).

## Discussion

This study was the first to report the association between asthma phenotype (as defined by BEC) and long-term adverse renal outcomes. Our data also suggested that renal progression in asthma patients was an independent factor for long-term mortality. Our results highlight the importance of aggressive and personalized management of asthma, which may potentially translate into improved outcomes in other organ systems including the kidneys and patient mortality. This also calls for regular monitoring of renal function in asthma patients and initiating reno-protective treatments should early signs of renal deterioration be detected.

Our findings suggested that patients with non-eosinophilic phenotype had higher risk of renal progression and more rapid decline in eGFR during subsequent follow-up when compared to those with eosinophilic phenotype. In this study, almost one-fifth of patients developed renal progression, and notably they had higher mortality compared with those without renal progression. The pathogenic link between asthma and adverse renal outcomes remains elusive. Here we speculated that chronic non-eosinophilic inflammation may contribute to renal progression in asthma as we detected significant associations between asthma phenotype and renal progression in this study. Non-eosinophilic asthma is well-reported to have distinct pathophysiology and clinical features when compared with eosinophilic asthma. Those with non-eosinophilic asthma are typically associated with a later age of onset, higher medication requirements, and significant symptomology^21–24^. Obese asthma, which is often associated with various metabolic complications, is also characterized with airway neutrophilia, and is more commonly non-eosinophilic^25^. Adult-onset asthma, which is more likely to be non-eosinophilic in phenotype, was also associated with higher odds of obesity, hypercholesterolemia, borderline high serum triglycerides than was childhood-onset asthma, after adjusted for age^26^. These metabolic disturbances in patients with non-eosinophilic asthma may predispose them to developing CKD and renal progression. Although lung function was reported to be better than in patients with eosinophilic asthma, patients with non-eosinophilic asthma also tend to have a poorer response to short-acting bronchodilators and ICS, which may further contribute to symptom burden^27,28^. The difficulty in asthma control, as reflected by higher medication requirements and less ICS sensitivity, may lead to hypoxaemia and a potential link to subsequent adverse renal outcomes. Those with non-eosinophilic asthma may also have airway neutrophilia with high interleukin-17 level^29,30^. These differences in pathogenic mechanisms and airway/systemic inflammation could contribute to the distinct risks of renal progression. For example, some of the inflammatory mediators and pathways that were reported to have possible riles in non-eosinophilic asthma, such as nucleotide-binding oligomerization domain (NOD)-like receptor family pyrin domain containing 3 (NLRP3) and neutrophil extracellular traps (NETs), were also reported^31,32^ to be associated with renal damage and dysfunction^33,34^.

The association between systemic inflammation and progressive CKD has been proposed in many studies^35–38^, which also contributes to impaired health status, reduced quality of life, and premature mortality^39^. For asthma, apart from airway inflammation, systemic inflammation is also present. The presence of systemic inflammation in asthma can contribute to the development of adverse outcomes in other organ systems, with cardiovascular events being reported. Our study is the first to suggest the link between inflammatory mechanism in asthma, adverse renal outcomes, and mortality. This highlights the importance of routine screening and monitoring of systemic complications including renal dysfunction among patients with asthma, in particular those with non-eosinophilic asthma. Compared with other systemic disease outcomes, early detection of kidney abnormalities by eGFR decline and proteinuria is relatively simple and timely interventions can significantly prevent progressive CKD and renal failure.

The proper and personalized management of asthma is important, as this will not only mitigate adverse respiratory outcomes but also adverse renal outcomes. Previous studies have reported that persistent asthma was associated with CKD^16^, and our current data further showed that non-eosinophilic asthma was associated with renal progression. These findings suggested that the early use of biologics should be considered in these at-risk patient subgroups. For instance, tezepelumab, a human monoclonal immunoglobulin G2-lambda antibody (AMG 157) that binds thymic stromal lymphopoietin (TSLP) and prevents its interaction with the TSLP receptor complex, has demonstrated efficacy on reducing asthma exacerbations among patients with BEC < 150 cells/mm^3^^40,41^. However, the safety of Tezepelumab among patients with CKD is yet to be determined. According to manufacturers’ labeling, there are no dosage adjustments provided. According to its pharmacokinetics, dose adjustment based on renal function is unlikely to be necessary as it is not renally eliminated^42^. The major limitations of this study were the single-centre nature, missing data during follow-up and the relatively small patient number included for analyzing clinical associations. By recruiting patients in QMH, a tertiary centre, more severe asthma patients or patients with more complicated medical history would be included, as they were those who need specialist clinic follow up. This could affect generalizability by having less mild asthma patients in the cohort. But our cohort does have patients with milder asthma which were only on ICS as maintenance therapy or even not on ICS, which are those with milder asthma. Having dedicated study to include more mild asthma patients will be ideal. Due to the variation in follow-up durations based on patients’ clinical conditions, the number of renal function tests and the timing of the tests were not unified in the cohort. Furthermore, longitudinal data on urinary protein excretion is sometimes lacking. Notwithstanding, all asthma patients followed up at our designated asthma clinic had comprehensive baseline lung function tests and clinical data collection, as well as standardized management for asthma, which allowed proper assessment of relationship with subsequent renal and mortality outcomes. In this retrospective study, not all patients have ANCA measured, which is one of the limitations we need to address. In the cohort, 147 patients had antineutrophil cytoplasmic antibodies (ANCA) measured, with 120 of them had a negative result, 26 with weak positive or atypical C-ANCA. None of the patients had eosinophilic granulomatosis with polyangiitis (EGPA) based on the 2022 American College of Rheumatology/European Alliance of Associations for Rheumatology Classification Criteria for Eosinophilic Granulomatosis with Polyangiitis^43^. We believe missing the cases with EGPA that caused renal progression to be unlikely in this study.

Our present findings provide further evidence of the adverse non-respiratory outcomes from asthma. The unfavorable renal outcomes as defined by renal progression is also related to mortality in patients with non-eosinophilic asthma. While the consequences of chronic renal insufficiency increase the burden of asthma patients, they also confer negative impacts on various clinical aspects, for example, limiting the options of pharmacotherapy for co-morbidities and asthma, aggravating cardiovascular complications, increasing healthcare service utilization and worsening patient survival. As such, timely initiation of preventive measures among patients with asthma, in particular non-eosinophilic asthma, should not be over-emphasized as the benefits may extend beyond optimizing respiratory conditions, but also other systemic morbidity, including renal and cardiovascular outcomes.

## Conclusions

CKD is prevalent in asthma patients, with non-eosinophilic asthma being an important risk factor for renal progression. Renal progression is also associated with mortality among patients with asthma.

## Supplementary Information

Supplementary Information.

## Data Availability

All data supporting the conclusion of this study is presented in this manuscript and no additional data will be provided. The data is available from author WC Kwok upon request.
