# Supplementary material for: Immunological phenotype in asthma and its impact on long-term renal outcomes
Source: Sci Rep. 2025 Oct 21;15:36538. doi: 10.1038/s41598-025-18035-5 (PMC12541048; doi:10.1038/s41598-025-18035-5)
Supplement: Supplementary file 1 — Supplementary Information. [file 41598_2025_18035_MOESM1_ESM.docx]

**Supplementary Tables**

**Table ST1. Baseline demographic and clinical characteristics of included patients based on renal progression**

|  | **No renal progression**  **(n = 429)** | | **Had renal progression**  **(n = 104)** | **Whole cohort**  **(n = 533)** | **p-values^** |
| --- | --- | --- | --- | --- | --- |
|  |  | |  |  |  |
| Age (years) | 60.3 ± 20.6 | | 68.9 ± 16.9 | 62.0 ± 20.2 | <0.001* |
| Gender |  | |  |  | 0.468 |
| Male | 165 (38.5 %) | | 36 (34.6%) | 201 (37.7%) |  |
| Female | 264 (61.5%) | | 68 (65.4%) | 332 (62.3%) |  |
| Smoking status |  | |  |  | 0.924 |
| Non-smoker | 344 (80.2%) | | 82 (78.8%) | 426 (79.9%) |  |
| Active smoker | 21 (4.9%) | | 6 (5.8%) | 27 (5.1%) |  |
| Ex-smoker | 64 (14.9%) | | 16 (15.4%) | 80 (15.0%) |  |
| BMI (kg/m^2^) | 24.2 ± 6.2 | | 25.1 ± 6.9 | 24.4 ± 6.3 | 0.31 |
| Co-morbidities |  | |  |  |  |
| Hypertension | 189 (44.1%) | | 64 (61.5%) | 253 (47.5%) | 0.001* |
| Diabetes mellitus | 82 (19.1%) | | 30 (28.8%) | 112 (21.0%) | 0.029* |
| Hyperlipidemia | 100 (23.3%) | | 31 (29.8%) | 131 (24.6%) | 0.167 |
| Atrial fibrillation | 34 (7.9%) | | 17 (16.3%) | 51 (9.6%) | 0.009* |
| Stroke/TIA | 46 (10.7%) | | 12 (11.5%) | 58 (10.9%) | 0.811 |
| Ischemic heart disease | 79 (18.4%) | | 220 (19.2%) | 99 (18.6%) | 0.848 |
| Bronchiectasis | 8 (3.7%) | | 21 (6.6%) | 29 (5.4%) | 0.156 |
| Atopic dermatitis | 147 (34.3%) | | 48 (46.2%) | 195 (36.6%) | 0.024* |
| Allergic rhinitis | 253 (82.3%) | | 89 (85.6%) | 442 (82.9%) | 0.423 |
| Medication |  | |  |  |  |
| ACEI/ARB | 159 (37.1%) | | 68 (65.4%) | 227 (42.6%) | <0.001* |
| Inhaled corticosteroid |  | |  |  | 0.395 |
| Low dose | 62 (14.5%) | | 21 (20.2%) | 83 (15.6%) |  |
| Medium dose | 122 (28.4%) | | 32 (30.8%) | 154 (18.9%) |  |
| High dose | 178 (41.5%) | | 38 (36.5%) | 216 (40.5%) |  |
| Long acting beta-agonists | 189 (44.1%) | | 56 (53.8%) | 245 (46.0%) | 0.072 |
| Long acting anti-muscarinic | 90 (21.0%) | | 32 (30.8%) | 122 (22.9%) | 0.033* |
| Leukotriene antagonists | 131 (30.5%) | | 34 (32.7%) | 165 (31.0%) | 0.670 |
| Theophylline | 63 (29.4%) | | 109 (34.2%) | 172 (32.3%) | 0.048* |
| Baseline FEV_1_ (L) | 1.82 ± 0.78 | | 1.51 ± 0.69 | 1.76 ± 0.77 | 0.003* |
| Baseline FEV_1_ (% predicted) | 79.6 ± 23.1 | | 73.2 ± 21.2 | 78.4 ± 22.9 | 0.051 |
| Baseline FEV_1_ to FVC (%) | 66.2 ± 14.3 | | 66.5 ± 11.8 | 66.3 ± 13.8 | 0.859 |
| Bronchodilator reversibility (%) | 10.4 ± 23.5 | | 9.05 ± 11.3 | 10.1 ± 21.6 | 0.565 |
| Bronchodilator reversibility (mL) | 130 ± 167 | | 107 ± 123 | 126 ± 159 | 0.277 |
| Baseline neutrophil count(x10^9^/L) | 4.81 ± 2.40 | 5.02 ± 2.47 | | 4.85 ± 2.41 | 0.417 |
| Baseline lymphocyte count (x10^9^/L) | 1.75 ± 0.70 | 1.76 ± 0.73 | | 1.75 ± 0.71 | 0.900 |
| Baseline eosinophil count (x10^12^/L) | 516 ± 677 | 445 ± 566 | | 502 ± 657 | 0.268 |
| Baseline HbA1C (%) | 4.75 ± 2.16 | 4.63 ± 2.33 | | 4.72 ± 2.19 | 0.711 |
| Serum low-density lipoprotein (mmol/L) | 2.01 ± 0.83 | 1.96 ± 0.84 | | 2.00 ± 0.83 | 0.619 |
| SII | 604 ± 606 | 575 ± 412 | | 598 ± 574 | 0.625 |
| NLR | 3.16 ± 3.68 | 3.16 ± 1.99 | | 3.16 ± 3.43 | 0.989 |
| Serum albumin level (g/L) | 41.38 ± 4.76 | 40.38 ± 4.02 | | 41.16 ± 4.63 | 0.070 |
| eGFR (mL/min/1.73m^2^), mean ±SD | 79.7 ± 27.5 | 77.7 ± 18.8 | | 79.3 ± 26.0 | 0.483 |
| Number of asthma exacerbation in past 12 months, mean ±SD | 0.52 ± 1.05 | 0.53 ± 0.93 | | 0.52 ± 1.03 | 0.958 |

BMI, body mass index, ACEI, angiotensin converting enzyme inhibitor; ARB, angiotensin receptor blockers; FEV1, forced expiratory volume in 1 second; FVC, forced vital capacity; eGFR, estimated glomerular filtration rates; TIA, transient ischaemic attack; SII, Systemic immune-inflammation index; NLR, Neutrophil-to-lymphocyte ratio

Data expressed as mean ± S.D.

**Table ST2. Tests for collinearity of the variables adjusted in multi-variable analysis**

| **Variables** | **Variance inflation factor (VIF)** |
| --- | --- |
| Age | 3.24 |
| Sex | 1.64 |
| Smoking status | 1.20 |
| BMI | 1.21 |
| Hypertension | 1.88 |
| Diabetes mellitus | 1.50 |
| Hyperlipidemia | 1.32 |
| Atrial fibrillation | 1.10 |
| Stroke/TIA | 1.11 |
| Ischemic heart disease | 1.28 |
| ACEI/ARB use | 2.08 |
| Baseline eGFR | 1.94 |
| Inhaled corticosteroid dose | 1.20 |
| Long acting beta-agonists | 1.14 |
| Long acting anti-muscarinic | 1.21 |
| Baseline FEV_1_ | 2.52 |

BMI, body mass index, ACEI, angiotensin converting enzyme inhibitor; ARB, angiotensin receptor blockers; FEV1, forced expiratory volume in 1 second; eGFR, estimated glomerular filtration rates; TIA, transient ischaemic attack
